# Ebola Virus Disease in the Era of One Health and Global Preparedness: Evolving Epidemiology, Genomic Surveillance, and Future Challenges

**DOI:** 10.3390/idr18050094

**Published:** 2026-08-28

**Authors:** Francesco De Maria, Francesco Branda, Ivailo Alexiev, Dong Keon Yon, Ayşe Banu Demir, Giancarlo Ceccarelli, Fabio Scarpa, Massimo Ciccozzi, Alessandro Russo

**Affiliations:** 1 Infectious and Tropical Diseases Unit, “Renato Dulbecco” Teaching Hospital of Catanzaro, 88100 Catanzaro, Italy; francescodemaria16@gmail.com (F.D.M.); a.russo@unicz.it (A.R.); 2 Unit of Medical Statistics and Molecular Epidemiology, Università Campus Bio-Medico di Roma, 00128 Rome, Italy; m.ciccozzi@unicampus.it; 3 National Reference Confirmatory Laboratory of HIV, National Center of Infectious and Parasitic Diseases (NCIPD), 1504 Sofia, Bulgaria; ivoalexiev@ncipd.org; 4 Center for Digital Health, Medical Science Research Institute, Kyung Hee University College of Medicine, Seoul 05278, Republic of Korea; yonkkang@gmail.com; 5 Department of Pediatrics, Kyung Hee University Medical Center, Kyung Hee University College of Medicine, Seoul 05278, Republic of Korea; 6 Department of Medical Biology, Faculty of Medicine, İzmir University of Economics, İzmir 35330, Türkiye; banu.demir@izmirekonomi.edu.tr; 7 Department of Public Health and Infectious Diseases, University Hospital Policlinico Umberto I, Sapienza University of Rome, 00185 Rome, Italy; giancarlo.ceccarelli@uniroma1.it; 8 Department of Biomedical Sciences, University of Sassari, 07100 Sassari, Italy; fscarpa@uniss.it

**Keywords:** Ebola virus disease, filovirus, One Health, genomic surveillance, ring vaccination, artificial intelligence, digital health, outbreak preparedness, Bundibugyo virus, health system resilience

## Abstract

Ebola virus disease (EVD) remains one of the most severe viral hemorrhagic fevers, with recurrent outbreaks challenging global healthcare systems. This narrative review traces the evolving epidemiology of EVD from the first recognized outbreaks in 1976 to the ongoing 2026 Bundibugyo virus public health emergency in the Democratic Republic of the Congo (DRC) and Uganda. Over nearly five decades, the recognized range of Ebola outbreak contexts and amplification mechanisms has broadened considerably: rural zoonotic spillovers remain the predominant mode of emergence, but the scale and reach of subsequent transmission increasingly depend on where introductions occur, on delays in detection, on population mobility, on ecological disruption, on armed conflict, on healthcare-associated transmission, and on viral persistence in survivors. Major advances in molecular epidemiology and genomic surveillance have improved outbreak investigation, enabling real-time transmission reconstruction and detection of survivor-linked resurgence. Vaccination, particularly ring vaccination with rVSV-ZEBOV, has shown high effectiveness against Zaire ebolavirus, yet vaccine equity gaps and the absence of licensed vaccines for Sudan virus and Bundibugyo virus remain critical vulnerabilities, though new candidate vaccines and therapeutics for Bundibugyo virus entered clinical evaluation in mid-2026. Artificial intelligence and digital technologies, including AI-assisted early warning systems, portable sequencing, drones, blockchain, and mobile health platforms, offer promising tools for outbreak preparedness, but robust evidence of their real-world operational impact during filovirus outbreaks remains limited, and their deployment in low-resource settings faces substantial barriers related to infrastructure, literacy, data costs, and governance. Integrated preparedness frameworks that combine ecological surveillance, resilient healthcare systems, community engagement, and international coordination under a One Health umbrella are increasingly viewed as a strategic necessity. The 2026 Bundibugyo outbreak reaffirms that despite decades of lessons, structural weaknesses in surveillance, response timeliness, and community trust continue to recur. Sustainable, multi-year financing, diversified vaccine platforms, regional manufacturing, and local co-design of digital tools are essential to translate lessons into lasting change. Preparedness is best understood as a continuous process rather than a reactive state.

## 1. Introduction

Ebola virus disease (EVD) remains one of the most severe viral hemorrhagic fevers and continues to represent a major challenge for global healthcare systems. According to the current International Committee on Taxonomy of Viruses (ICTV) taxonomy, the causative agents are classified within the genus Orthoebolavirus and include species such as *Orthoebolavirus zairense* (formerly Zaire ebolavirus), *Orthoebolavirus sudanense* (Sudan virus), and *Orthoebolavirus bundibugyoense* (Bundibugyo virus). Under current ICTV nomenclature, the virus name (e.g., Ebola virus, EBOV) and the species name (e.g., *Orthoebolavirus zairense*, formerly referred to as “Zaire ebolavirus”) are formally distinct; in this review we use the established virus names (Ebola virus/EBOV, Sudan virus, Bundibugyo virus) for readability, following standard virological convention, while noting the corresponding formal species designations here. Since its first identification in 1976 in what was then Zaire (now the Democratic Republic of the Congo, DRC) and Sudan, the recognized scope of Ebola has progressively broadened from that of a geographically confined zoonotic infection to a public health threat with wide-ranging epidemiological, political, and societal implications [1]. The 2013–2016 West African epidemic, caused by the Zaire ebolavirus species, marked a critical turning point in this broadening picture. That outbreak, which involved Guinea, Sierra Leone, and Liberia, resulted in more than 28,000 confirmed, probable, and suspected cases and over 11,000 deaths, clearly demonstrating how rapidly localized outbreaks can overwhelm fragile healthcare infrastructures and evolve into international public health emergencies when introductions occur in mobile, densely populated settings [1]. The epidemic exposed profound weaknesses in global surveillance systems, laboratory capacity, infection prevention and control (IPC) measures, and international coordination mechanisms while also highlighting the consequences of delayed response, community mistrust, and unsafe burial practices [1].

More recent outbreaks have further underscored the persistent threat posed by filoviruses, particularly in settings affected by armed conflict, forced displacement, limited surveillance capacity, and fragile healthcare systems. The 2018–2020 outbreak in eastern DRC, for instance, occurred in an active conflict zone and required unprecedented public health measures, the first widespread deployment of rVSV-ZEBOV, initially under expanded/compassionate access prior to subsequent formal licensure (FDA approval in December 2019, followed by DRC/African licensure in early 2020), under a ring vaccination strategy [2,3]. Similarly, the 2022 Sudan virus outbreak in Uganda reminded the global community that vaccine availability remains unequal across different ebolavirus species, as no licensed vaccine against Sudan virus was available at that time [3]. In parallel, the declaration of a public health emergency of international concern (PHEIC) linked to the 2026 outbreaks of Bundibugyo virus disease (a species first described in 2007, for which no vaccine was licensed at outbreak onset) in Central Africa, with the emergency declared in May 2026, has once again highlighted the continuing importance of Ebola preparedness within broader global health security agendas [4,5,6]. The ongoing 2026 outbreak involves a virus species for which no licensed vaccine or therapeutic exists, underscoring the need for diversified preparedness strategies that extend beyond the historical focus on Zaire ebolavirus [4]. These successive events demonstrate that Ebola is not a problem of the past but a recurring and evolving threat that demands sustained attention.

Over the last decade, the understanding of Ebola epidemiology has evolved substantially. Ebola outbreaks are no longer viewed simply as isolated zoonotic spillover events, but rather as the result of multiple interconnected ecological, biological, and societal drivers whose relative contribution varies by setting. Environmental disruption, population mobility, healthcare-associated transmission, sociopolitical instability, and viral evolution have all been associated with outbreak emergence and amplification in different contexts [2,7,8,9]. For example, financial and trade networks may indirectly influence land-use change and wildlife exploitation, and have been associated with increased spillover risk [7]. Likewise, the convergence of population growth, agricultural expansion, and wildlife habitat loss has been shown to correlate with filovirus outbreak hotspots in sub-Saharan Africa [8]. At the same time, evidence supporting the role of bats and other wildlife reservoirs, together with the documented impact of deforestation, mining activities, urban expansion, and climate-sensitive environmental changes, has strengthened interest in One Health approaches that integrate human, animal, and environmental health perspectives [8,10,11,12]. The DRC, which has experienced more Ebola outbreaks than any other country, exemplifies the complex interplay between ecological disruption (e.g., artisanal mining encroaching on forest habitats), population displacement due to armed conflict, and weak healthcare systems [3,11]. These integrated frameworks are increasingly viewed not only as conceptual tools but as practical necessities for epidemic prevention, early warning, and long-term preparedness [10,12].

Major advances in molecular epidemiology and genomic surveillance have also improved the investigation of Ebola outbreaks. Real-time sequencing technologies, including both short-read and emerging long-read platforms, now make it possible to reconstruct transmission chains with high resolution, identify patterns of viral persistence, distinguish novel zoonotic spillover events from recrudescence associated with survivors, and monitor viral evolution during an outbreak [13,14,15,16]. During the 2018–2020 outbreak in eastern DRC, real-time genomic data were used to confirm epidemiological links, rule out alternative chains of transmission, and directly inform public health decision-making, including vaccine allocation and the expansion of vaccination to identified high-risk groups [17]. In a landmark study of the 2013–2016 West African epidemic, genomic analyses quantified the added value of viral sequencing for inferring who infected whom, demonstrating that even partial genomic data can substantially reduce uncertainty in transmission network reconstruction [13]. More recently, the 2021 Ebola resurgence in Guinea represented a particularly important epidemiological signal. Genomic analyses revealed that the outbreak strain was closely related to viruses from the 2013–2016 West African epidemic rather than to a new zoonotic introduction, challenging the assumption that outbreaks arise exclusively from new animal-to-human transmission events and suggesting a role for long-term viral persistence in some instances of epidemic re-emergence [9,15]. This finding has implications for survivor follow-up programmes, discussed in detail in Section 5, as viral persistence has been documented in immune-privileged sites such as the testes, central nervous system, and ocular tissues months or even years after clinical recovery [9,15].

Important progress has also been achieved in outbreak response strategies, including vaccination campaigns (notably ring vaccination using rVSV-ZEBOV), rapid diagnostic platforms (including GeneXpert-based assays for decentralised testing), IPC measures, digital surveillance systems (such as mobile health applications for contact tracing), epidemiological modelling for forecasting and resource allocation, and integrated preparedness frameworks that combine surveillance, laboratory networks, and community engagement [18,19,20]. The COVID-19 pandemic, while distinct in its transmission dynamics, also provided lessons transferable to Ebola preparedness, particularly regarding the value of digital tools, genomic surveillance infrastructure, and the need for flexible healthcare systems [18]. However, several major challenges remain unresolved: global inequities in vaccine access, with low-income countries often facing delays and shortages; the spread of misinformation and conspiracy theories that undermine public health interventions; the complexity of mounting responses in active humanitarian crises and conflict zones; the erosion of community trust following decades of marginalisation or poorly handled past outbreaks; and the structural fragility of healthcare systems in resource-limited settings, which affects not only outbreak response but also routine health service delivery [21,22,23,24,25,26]. These challenges are structural rather than merely operational, and they persist across outbreaks despite repeated lessons learned.

This narrative review was developed through a targeted search of PubMed/MEDLINE, Scopus, and Google Scholar, complemented by outbreak reports and situation updates from the World Health Organization, the Africa Centres for Disease Control and Prevention, and the US Centers for Disease Control and Prevention. Search strategies incorporated the terms “Ebola”, “Ebola virus disease”, “filovirus”, and individual species names in combination with keywords such as “epidemiology”, “genomic surveillance”, “One Health”, “vaccination”, “outbreak response”, and ”preparedness”. Sources published between 1976 and early August 2026 were considered, with particular emphasis on the recent peer-reviewed literature and authoritative public health documents; given the pace of the ongoing 2026 outbreak, outbreak-specific figures in Section 8.3 reflect the most recent situation reports available at the time of this revision and should be read as a snapshot rather than a final outbreak size. As this review follows a narrative rather than a systematic approach, no predefined protocol, quantitative meta-analysis, or formal risk-of-bias assessment was performed. Instead, sources were selected to capture key developments, emerging trends, and major challenges shaping the contemporary landscape of Ebola virus disease epidemiology, surveillance, prevention, and outbreak preparedness.

To provide a comprehensive perspective, this review is organized into several thematic sections. We first examine the historical evolution of Ebola outbreaks, from the 1976 index cases to the ongoing 2026 Bundibugyo public health emergency, highlighting key epidemiological characteristics and preparedness lessons (Section 2). Next, we analyse the ecological and zoonotic drivers of spillover events, including deforestation, mining, climate change, and the role of wildlife reservoirs, and discuss how One Health approaches can integrate human, animal, and environmental surveillance to reduce emergence risk (Section 3). We then explore transmission dynamics, showing how urbanization, population mobility, healthcare-associated amplification, and conflict can transform a localized introduction into an urban or international event (Section 4). Subsequently, we review advances in molecular epidemiology and genomic surveillance (Section 5), assess vaccination strategies and outbreak containment (Section 6), and examine future challenges and global preparedness, with a dedicated subsection on artificial intelligence and emerging technologies that explicitly distinguishes demonstrated operational capability from computational proof-of-concept (Section 7). A critical discussion follows, synthesising lessons from COVID-19 for Ebola preparedness, barriers to digital technology implementation, and the 2026 Bundibugyo outbreak as a contemporary test of global response capacity (Section 8). Finally, we draw conclusions and outline actionable priorities for policy makers, researchers, and healthcare systems (Section 9).

## 2. Historical Evolution of Ebola Outbreaks

The first recognized outbreaks of Ebola virus disease occurred in 1976 in two distinct locations: one in Sudan (near the town of Nzara, now South Sudan) and another in Zaire (now the DRC), near the Ebola River from which the virus takes its name [27]. These early epidemics highlighted the extreme clinical severity of filovirus infections, with case-fatality rates (CFR) approaching 53% in Sudan and 88% in Zaire, established Ebola virus as a major emerging pathogen, and revealed the potential for nosocomial amplification: in Zaire, transmission was amplified by the reuse of contaminated needles and syringes in missionary hospitals [27]. At that time, most outbreaks appeared geographically limited, typically involving remote rural areas characterized by poor healthcare infrastructure and relatively low population mobility.

For nearly two decades after 1976, Ebola was largely perceived as a sporadic zoonotic disease confined to Central Africa, with only occasional small outbreaks, such as the 1995 epidemic in Kikwit, DRC. The Kikwit outbreak, caused by Zaire ebolavirus, was the first major urban-associated Ebola outbreak, with 315 cases and 250 deaths (CFR 79%), and demonstrated how delayed outbreak recognition and inadequate IPC measures in healthcare settings can transform a localized spillover into a large-scale urban crisis [28]. The response to Kikwit also marked the first use of a field laboratory for real-time diagnosis and the implementation of barrier nursing techniques, laying the groundwork for modern outbreak containment strategies [19,28].

The 2000–2001 outbreak in Gulu, Uganda, caused by Sudan virus, represented another important milestone. With 425 cases and a case-fatality rate of approximately 53%, it was the largest Sudan virus outbreak recorded at that time and demonstrated the value of enhanced surveillance, contact tracing, and international coordination [29]. Gulu benefited from a comparatively rapid international response, including support from the World Health Organization (WHO), the US Centers for Disease Control and Prevention (CDC), and Médecins Sans Frontières (MSF), which helped limit geographic spread despite the absence of a licensed vaccine or specific therapeutics for Sudan virus [29], demonstrating that well-coordinated public health responses can control filovirus epidemics in resource-constrained settings when implemented rapidly.

Transmission patterns changed markedly during the 2013–2016 West African epidemic, the largest Ebola outbreak ever recorded, which reshaped the global understanding of filovirus epidemiology [1]. Unlike previous outbreaks, transmission spread extensively through densely populated urban centers and across international borders, involving Guinea, Sierra Leone, and Liberia. The epidemic exposed major weaknesses in surveillance systems, laboratory capacity, infection prevention infrastructure, and international preparedness mechanisms, and illustrated the consequences of delayed global response: the outbreak had already been smouldering for months before formal recognition, allowing exponential growth that ultimately overwhelmed local and international capacities [1]. Fear, misinformation, unsafe burial practices, mistrust toward institutions, and healthcare-associated transmission all contributed to sustaining transmission and complicating containment [30,31], while intense population mobility and cross-border movement facilitated regional dissemination. The epidemic ultimately caused over 28,000 cases and more than 11,000 deaths, with estimated economic losses of US$3.6 billion annually at its peak [1].

Subsequent outbreaks in the DRC and Uganda further confirmed that Ebola response becomes considerably more difficult in settings affected by conflict, political instability, and humanitarian crises [32]. The 2018–2020 outbreak in eastern DRC, which occurred in an active war zone with widespread displacement, required public health innovations including the first routine use of the rVSV-ZEBOV vaccine under a ring vaccination strategy, real-time deployment of mobile laboratories and genomic surveillance, and the integration of social anthropologists to address community mistrust [14,20,23]. The 2022 Sudan virus outbreak in Uganda, by contrast, illustrated that vaccine equity remains incomplete: although a highly effective vaccine exists for Zaire ebolavirus, no licensed vaccine was available for Sudan virus at that time, forcing reliance on public health measures alone [32,33].

More recently, evidence from several outbreaks has qualified one of the traditional assumptions of filovirus epidemiology: that epidemics arise exclusively from new zoonotic spillover events. Molecular investigations and genomic analyses have shown that some outbreaks may instead be linked to viral persistence in survivors, with recrudescence occurring months or even years after the initial infection [13,14,15,34,35]. The 2021 Guinea outbreak represented a particularly important example: genomic sequencing revealed that the responsible strain was closely related to viruses circulating during the 2013–2016 West African epidemic rather than to a new zoonotic introduction, suggesting a survivor-linked origin (see Section 5 for a fuller discussion of the genomic evidence and its implications for long-term survivor monitoring) [15].

These observations indicate that Ebola virus disease is best understood not as a uniformly episodic rural zoonosis, but as a threat whose recognized range of contexts has broadened to include ecological disruption, urbanization, population mobility, sociopolitical instability, healthcare fragility, and the long-term consequences of viral persistence, with rural zoonotic spillover remaining the predominant mode of initial emergence. As summarized in Table 1, the major Ebola outbreaks discussed in this review, from the 1976 index cases to the ongoing 2026 Bundibugyo public health emergency, reveal how transmission patterns and epidemiological complexity have varied by setting, and the corresponding lessons learned for global preparedness.

## 3. Ecological and Zoonotic Drivers

The emergence and re-emergence of Ebola virus disease are associated with ecological and environmental changes that shape interactions between humans, wildlife reservoirs, and surrounding ecosystems. Although bats, particularly species of the families Pteropodidae (fruit bats) and Molossidae (insectivorous bats), are widely considered plausible natural reservoirs for filoviruses, the precise mechanisms driving spillover events remain only partially understood [10,12]. Serological and molecular surveys have detected Ebola virus antibodies and RNA in several bat species across sub-Saharan Africa, but direct isolation of infectious virus from naturally infected bats has rarely been achieved, leaving the definitive reservoir status unconfirmed [39,40]. Experimental infections have shown that bats can support Ebola virus replication without developing disease, consistent with a reservoir host profile [41].

Over recent decades, progressive human encroachment into forest ecosystems has altered the human–wildlife interface. Deforestation, mining activities, agricultural expansion, biodiversity loss, and ecosystem fragmentation have each been associated with increased opportunities for zoonotic transmission [2,7,8]. In Central and West Africa, logging roads and mining camps provide access to previously remote forest areas, bringing humans into closer contact with wildlife and their habitats; hunting and consumption of bushmeat, including non-human primates and bats, represents a plausible direct pathway for spillover [42]. Mining operations and conflict-related population displacement have further disrupted ecological balances in several African regions, and have been associated with changes in spillover dynamics [11]; artisanal gold and coltan mining in eastern DRC, for example, has been linked with both deforestation and human displacement, creating conditions that favour contact between miners and reservoir species [11].

Environmental and climate-sensitive factors may also be associated with Ebola epidemiology. Variations in rainfall, temperature, habitat distribution, and food availability have been associated with reservoir behavior, wildlife migration patterns, and viral circulation within animal populations [2]. Seasonal patterns have been observed in some Ebola outbreaks, with peaks often following dry seasons or periods of food scarcity that may drive bats closer to human settlements [43]. A direct causal link between climate change and filovirus emergence remains difficult to establish, and the available evidence is associative rather than mechanistic. Broader cross-species viral-sharing models project that climate-driven shifts in mammalian host ranges could alter contact patterns between wildlife species, and potentially between wildlife and humans, in sub-Saharan Africa [44,45]; this modelling addresses mammalian viral sharing in general rather than filovirus spillover specifically, and whether or how it translates into changes in Ebola-specific spillover risk in particular regions has not been directly modelled. We therefore treat any geographic projection of future Ebola risk as hypothesis-generating rather than established, and highlight it as a priority for dedicated Ebola-specific climate modelling.

Current evidence suggests that Ebola emergence rarely depends on a single ecological trigger alone. Spillover events likely arise from the interaction of multiple factors, including environmental disruption, wildlife ecology, human behavioral patterns, population mobility, sociopolitical instability, and healthcare system fragility [8,9,35], which may partly explain the heterogeneity observed across outbreaks in transmission dynamics, epidemic size, and geographic spread. For instance, the 2013–2016 West African epidemic has been associated with a combination of deforestation, increased bat populations in fragmented habitats, and high population mobility, whereas the 2022 Sudan virus outbreak in Uganda occurred in a context of recent land-use change and wildlife encroachment [8,32].

These observations have implications for preparedness strategies. Outbreak-response models focused primarily on human-to-human transmission are unlikely to be sufficient as a standalone strategy; future preparedness efforts will probably require stronger integration between ecological surveillance, wildlife monitoring, genomic epidemiology, mathematical modelling, and community-based preparedness systems [20,21,35]. Ecological surveillance involves monitoring wildlife mortality events, testing bushmeat, and tracking changes in land use and climate that may help anticipate spillover risk. Wildlife monitoring programmes, such as the US Agency for International Development’s PREDICT project, have contributed to building capacity for wildlife surveillance; PREDICT-affiliated researchers directly demonstrated the feasibility of detecting novel filoviruses in bat populations, including the discovery of Bombali virus in Sierra Leonean bats [46], though such programmes require sustained funding and cross-sectoral collaboration that remain challenging in resource-limited settings. In this context, One Health approaches are increasingly viewed not only as conceptual frameworks but also as practical tools for epidemic prevention, surveillance, and long-term preparedness.

**Figure 1:**
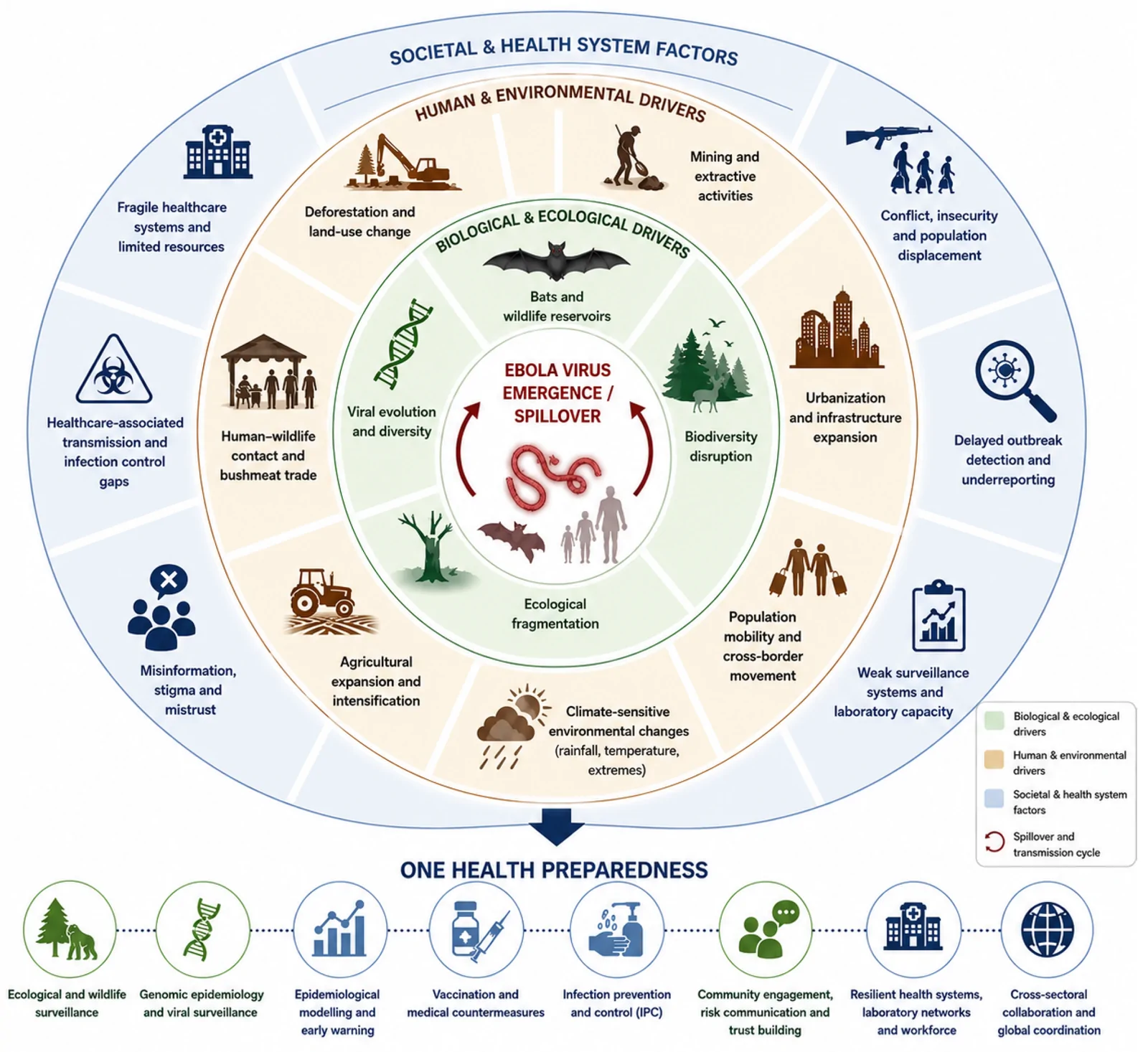
Conceptual framework of Ebola virus emergence and One Health preparedness.

Figure 1 provides a conceptual framework illustrating the multifactorial drivers of Ebola virus emergence and outbreak amplification through a One Health perspective, organised into three interconnected domains: (i) biological and ecological determinants (wildlife reservoirs, biodiversity disruption, viral evolution, ecological fragmentation); (ii) human and environmental drivers (deforestation, mining, urbanisation, climate-sensitive environmental changes, population mobility); and (iii) societal and healthcare-related factors (fragile healthcare systems, healthcare-associated transmission, misinformation and mistrust, armed conflict, delayed outbreak detection). The lower portion of the figure summarises the major components of integrated One Health preparedness strategies. Together, these components illustrate that Ebola emergence is not a linear process but a system in which ecological, social, and healthcare-system factors interact to shape spillover risk, outbreak amplification, and response effectiveness.

A more detailed breakdown of these drivers, categorised by mechanism, epidemiological consequence, and preparedness implication, is summarized in Table 2.

## 4. Transmission Dynamics

The transmission dynamics of Ebola virus disease vary considerably across settings, reflecting differences in urbanization, population mobility, healthcare infrastructure, and outbreak response capacity [1]. Earlier outbreaks, from 1976 through the early 2000s, were generally characterized by relatively short transmission chains occurring in isolated rural communities with limited geographic spread; the 1976 Yambuku outbreak in Zaire, for example, despite a CFR of 88%, remained confined to a radius of approximately 70 kilometres and was controlled largely through isolation of affected villages and discontinuation of needle reuse [27]. This pattern of contained rural transmission has not disappeared and remains the more common outcome; it now coexists with outbreaks in which urbanization, mobility and healthcare-associated transmission drive much larger amplification when introductions occur in densely populated and highly interconnected environments.

The 2013–2016 West African epidemic illustrated this latter pattern most starkly. Unlike any previous outbreak, transmission expanded extensively within urban settings, including the capital cities of Conakry, Freetown, and Monrovia, and across international borders, involving Guinea, Sierra Leone, and Liberia [1]. High population density, delayed case recognition (the outbreak likely circulated undetected for about three months before being formally declared), inadequate infection prevention measures in overstretched healthcare facilities, and intense regional mobility all contributed to rapid dissemination [1]. Mathematical modelling studies have since shown that each week of delay in implementing control measures during the exponential growth phase is associated with a substantial increase in final epidemic size, with later intervention requiring far greater resources to achieve the same containment effect [49,50]. These events demonstrated that, where such conditions are present, Ebola can also become an urban and international threat rather than remaining confined to remote rural areas.

Healthcare-associated transmission has been one of the major drivers of epidemic amplification in several outbreaks. Limited IPC infrastructure, shortages of personal protective equipment (PPE), delayed isolation, and inadequate laboratory capacity have repeatedly facilitated nosocomial transmission involving both patients and healthcare workers [19,28]. During the 1995 Kikwit outbreak, approximately one-third of cases were estimated to be healthcare-associated, primarily due to reuse of contaminated needles and lack of barrier nursing [28]; during the 2018–2020 eastern DRC outbreak, dozens of healthcare workers were infected, and several treatment centres became epicentres of transmission before IPC measures were strengthened [19]. These recurring patterns underscore the need for IPC capacity building as a core component of healthcare system resilience, with sustained investment required even during inter-epidemic periods.

Humanitarian crises and armed conflicts add further complexity. Insecurity may restrict access to affected communities, interrupt vaccination campaigns, compromise contact tracing, displace populations, and weaken trust in healthcare institutions [11,23,30]. During the 2018–2020 outbreak in eastern DRC, ongoing violence from over 130 armed groups, together with population displacement affecting more than 1.5 million people, substantially complicated containment strategies and contributed to prolonged transmission chains [30]. The outbreak lasted nearly two years, the second longest in history, despite the availability of a highly effective vaccine, illustrating how conflict dynamics can override the benefit of biomedical tools [23].

Social and behavioral determinants remain important. Fear, misinformation, mistrust toward authorities, and unsafe burial practices have repeatedly influenced outbreak trajectories and undermined public health interventions [24,25,31]. Funeral- and burial-associated transmission was a major driver of the 2013–2016 West African epidemic, and safe and dignified burial practices became a central pillar of outbreak control [31]; culturally adapted risk communication and sustained community engagement are now widely recognised as essential components of effective outbreak preparedness and response; interventions that involve local leaders, traditional healers, and religious authorities in designing safe burial protocols have been shown to increase acceptance and reduce transmission [25].

**Figure 2:**
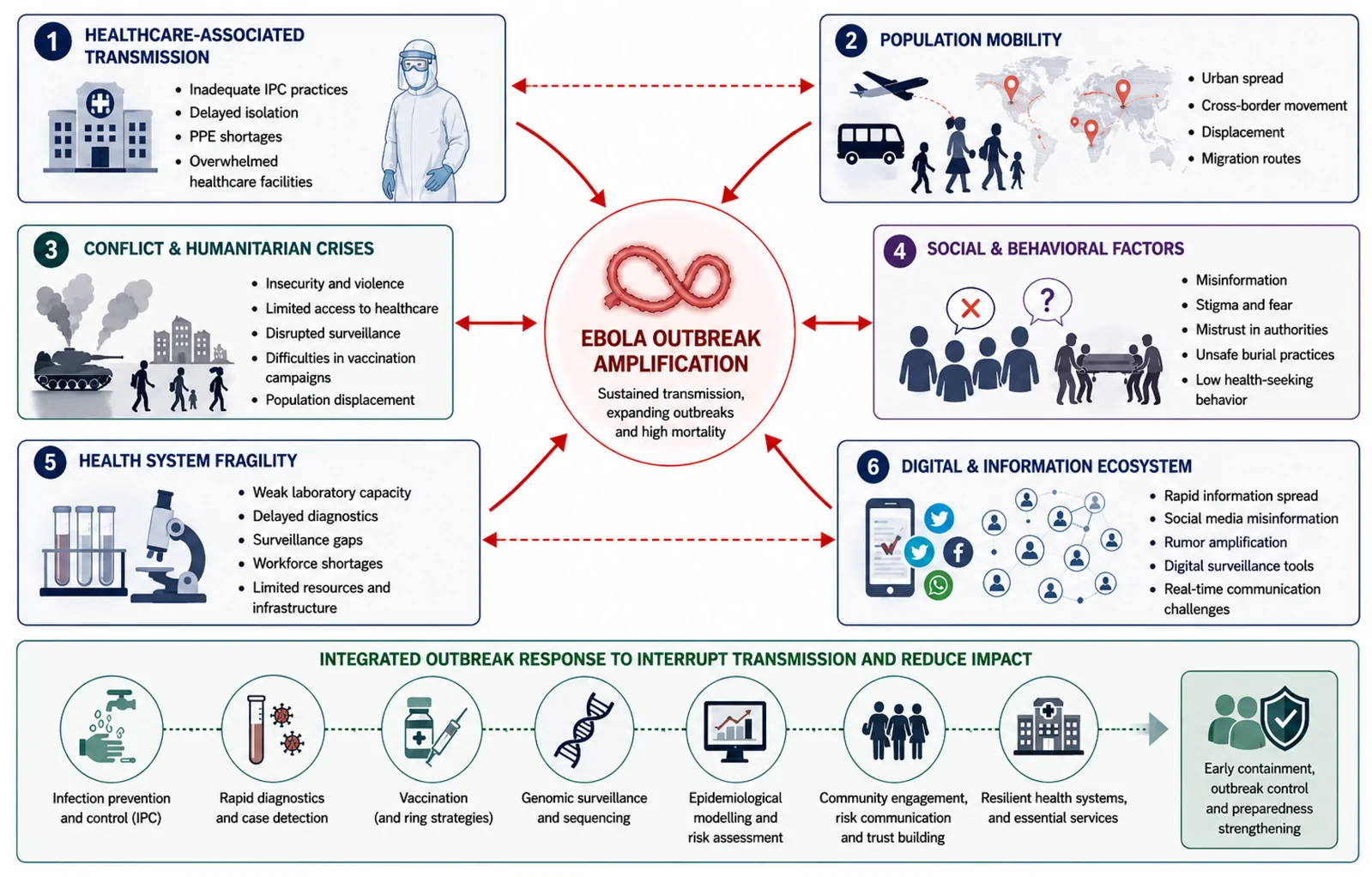
Interconnected drivers of transmission amplification and core components of outbreak response. Solid arrows indicate direct causal pathways (e.g., inadequate IPC directly increases nosocomial transmission); dashed arrows represent indirect or feedback effects (e.g., misinformation via digital ecosystems exacerbates mistrust, reducing adherence to control measures).

Modern Ebola epidemiology therefore reflects far more than viral transmission alone: outbreak propagation depends on the interaction between biological, ecological, political, social, and healthcare-related factors, including mobility networks, healthcare system fragility, conflict settings, digital information ecosystems, and institutional trust. Effective outbreak control accordingly requires integrated multidisciplinary strategies extending beyond purely biomedical interventions [3,20,23]. Figure 2 summarises these drivers of transmission amplification alongside the principal components of integrated outbreak response.

## 5. Molecular Epidemiology and Genomic Surveillance

Advances in molecular epidemiology and genomic surveillance have substantially improved the understanding of Ebola virus transmission and outbreak evolution. During the 2013–2016 West African epidemic, real-time genomic sequencing was deployed for the first time during an ongoing Ebola outbreak, demonstrating its potential to inform public health actions [51]. Subsequent outbreaks in the DRC and Uganda have progressively integrated genomic surveillance as a routine component of outbreak investigation, allowing reconstruction of transmission chains, identification of viral introductions, monitoring of viral evolution, and differentiation between zoonotic spillover events and sustained human-to-human transmission [13,14].

Real-time genomic surveillance enables outbreak dynamics to be investigated with a level of precision that was previously unattainable. Sequencing data can help identify transmission clusters that are epidemiologically cryptic, clarify links between geographically dispersed cases, and support public health decision-making when integrated with epidemiological investigations and mathematical modelling [13,20]. During the 2018–2020 outbreak in eastern DRC, genomic data were used to confirm or refute suspected epidemiological links, inform vaccine allocation and the expansion of vaccination to identified high-risk groups, and rule out separate concurrent spillover events [17]. Compared with earlier outbreaks, where investigations relied primarily on contact tracing and clinical surveillance, genomic epidemiology has markedly improved the understanding of how outbreaks emerge, propagate, and terminate.

Genomic analyses have also clarified mechanisms of viral persistence and recrudescence. Retrospective investigations from outbreaks in the DRC revealed complex transmission patterns involving both local amplification and interconnected outbreak networks spanning multiple health zones and, in some cases, international borders [14,52]. Genomic analyses conducted during the 2021 Guinea outbreak suggested that long-term viral persistence in survivors may contribute to epidemic re-emergence, qualifying the assumption that all outbreaks originate exclusively from novel zoonotic spillover events [9,15]; in that case, the recovered viral genome was nearly identical to sequences from the 2013–2016 West African epidemic, ruling out a new animal introduction and pointing to a survivor-linked resurgence [15].

This emerging paradigm carries implications for surveillance and preparedness. Persistent viral reservoirs within immune-privileged sites, including the testes, central nervous system, and ocular tissues, may allow Ebola virus to persist long after apparent clinical recovery [34,35]. Studies have documented Ebola virus RNA in semen for up to 500 days after acute infection, with rare confirmed cases of sexual transmission [34], and recrudescence from the central nervous system has been implicated in late relapses with meningoencephalitis [35]. These findings have led to the establishment of long-term survivor monitoring programmes, including semen testing and counselling, although implementation in resource-limited settings remains challenging.

The growing integration of genomic surveillance into outbreak-response frameworks represents a major advance in modern filovirus preparedness. Portable sequencing technologies, such as Oxford Nanopore MinION, have enabled field-deployable genomic surveillance in remote outbreak settings, with turnaround times from sample collection to sequence results reduced to less than 48 h [51]. Long-read sequencing platforms may further improve rapid outbreak characterization by resolving difficult-to-sequence genomic regions and by providing more complete viral genomes from low-titre clinical samples, which are common in field settings [16]. Nevertheless, major disparities in sequencing infrastructure, laboratory capacity, bioinformatics expertise, and access to genomic technologies persist across many endemic regions; the cost of sequencing, the need for stable electricity and internet connectivity, and the shortage of trained bioinformaticians remain substantial barriers to equitable implementation [53].

Overall, molecular epidemiology has changed the contemporary interpretation of Ebola virus disease. Outbreaks are increasingly understood to reflect interactions involving ecological emergence, viral evolution, survivor-associated persistence, human mobility patterns, and healthcare system vulnerabilities [9,13,14,15,20]. The ability to sequence and analyse viral genomes in near real time has moved outbreak investigation from a reactive, descriptive exercise toward a more proactive, hypothesis-driven discipline. However, the full potential of genomic surveillance will only be realised when it is systematically integrated into routine public health surveillance in high-risk regions, supported by sustainable funding, workforce training, and open data-sharing frameworks.

## 6. Vaccination and Outbreak Containment

Vaccination has progressively become one of the central pillars of modern Ebola outbreak response strategies. Prior to 2015, outbreak control relied exclusively on classic public health measures, i.e., case isolation, contact tracing, safe burials, and community engagement, which, although effective when rigorously implemented, often proved insufficient in densely populated or conflict-affected settings [1].

The pivotal breakthrough came with the rVSV-ZEBOV vaccine (Ervebo^®^), a recombinant vesicular stomatitis virus vector expressing the Ebola virus (EBOV) glycoprotein from the Zaire ebolavirus species. A landmark cluster-randomised trial conducted during 2015, within the West African epidemic (the Ebola Ça Suffit! trial), demonstrated 100% efficacy (95% CI 74.7–100%) when administered as a ring vaccination strategy, with no confirmed cases among vaccinated individuals 10 days or more after immunisation [54]. This trial, coordinated by WHO and partners, moved from phase 1 trials to proof-of-concept efficacy data in less than 12 months.

Ring-vaccination strategies using rVSV-ZEBOV have since been deployed during outbreaks in the DRC and elsewhere, demonstrating effectiveness in real-world emergency settings [55]. During the 2018–2020 eastern DRC outbreak, over 300,000 people were vaccinated, including frontline healthcare workers and contacts of confirmed cases; an early WHO preliminary analysis estimated approximately 97.5% effectiveness, whereas a later retrospective test-negative study estimated 84% effectiveness from ≥10 days post-vaccination [56,57]. These experiences showed that rapid vaccine deployment is feasible even in highly complex epidemics, including active conflict zones, and yielded operational lessons including the use of mobile vaccination teams, community mobilisers, and decentralised cold chain logistics (e.g., Arktek passive cooling devices) that enabled delivery in remote areas without reliable electricity [58].

Despite these advances, important challenges remain. Access to Ebola vaccines continues to be marked by global inequities, particularly in low-resource regions where outbreaks are most likely to occur [22,26]. The rVSV-ZEBOV vaccine is licensed for disease caused by Zaire ebolavirus; WHO does not currently recommend it for outbreaks caused by Sudan virus or Bundibugyo virus, since it lacks demonstrated clinical efficacy against these species. It is important to note that this reflects an absence of proven cross-protective efficacy in humans rather than established evidence of zero biological cross-reactivity; WHO’s Filovirus Research and Development Roadmap has commissioned a formal review of Ervebo’s cross-immunity potential and of prime-boost strategies against Bundibugyo virus [59]. Logistical barriers, including ultra-cold chain requirements for some formulations (rVSV-ZEBOV is stable at −80 °C to −60 °C per FDA prescribing information, although a refrigerated version has since been developed), manufacturing limitations, and unequal global distribution systems, may delay vaccine availability during emergencies [60].

Vaccine acceptance also remains a critical determinant of outbreak containment. Fear, misinformation, mistrust toward institutions, and previous negative experiences with healthcare systems may strongly influence vaccine uptake [25,55]. During the 2018–2020 DRC outbreak, pockets of vaccine hesitancy were documented, particularly in areas affected by prolonged conflict and where rumours circulated about the vaccine being used for population control [61]. Successful vaccination strategies therefore require not only vaccine availability but also sustained community engagement, transparent communication, and culturally adapted implementation; the integration of social scientists and anthropologists into vaccination teams, pioneered during the West African epidemic, has become standard practice for addressing hesitancy [61].

Ebola vaccination programs also provided broader lessons extending beyond filovirus outbreaks. Experiences gained during Ebola vaccine development and deployment informed several approaches later adopted during the COVID-19 pandemic, including emergency use authorisation pathways, adapted ring-vaccination strategies, and international coordination mechanisms such as WHO’s Strategic Advisory Group of Experts (SAGE) recommendations and the WHO Emergency Use Listing (EUL) regulatory procedure [62]; the concept of “ring vaccination”, originally developed for smallpox eradication and refined for Ebola, was explored for COVID-19 in selected settings [62].

Filovirus vaccines are increasingly viewed not simply as outbreak-control tools, but as strategic components of broader preparedness platforms for emerging infectious diseases and future pandemic threats [36,63,64]. This reflects growing recognition that future preparedness will require scalable vaccine technologies, flexible manufacturing systems, equitable global distribution mechanisms, and stronger regional preparedness capacities, including local vaccine production in Africa [65,66,67]. WHO’s R&D Blueprint has listed filoviruses as priority and prototype pathogens, and ongoing research aims to develop pan-filovirus vaccines that could provide cross-protection against multiple ebolavirus and marburgvirus species [64].

These efforts are now being coordinated through WHO’s Filovirus Research and Development Roadmap, developed by the Filovirus Clinical Research Coordination (CORC) group under the R&D Blueprint and building on the 2021–2031 WHO-AFIRM strategy. The roadmap identifies Ebola virus and Marburg virus as R&D Blueprint priority and prototype pathogens, sets out research priorities spanning vaccines, therapeutics, and diagnostics for all ebolavirus species, and has commissioned the cross-immunity review noted above. During the 2026 outbreak, this roadmap has been cited by WHO and Africa CDC as a basis for accelerating R&D investment in non-Zaire ebolavirus species [59].

Important uncertainties remain regarding the durability of vaccine-induced protection, cross-protection against different filoviruses, vaccine implementation in humanitarian settings, and optimal deployment strategies (ring versus mass vaccination) during rapidly evolving outbreaks. The 2026 Bundibugyo outbreak, for which no species-specific vaccine was licensed at onset, prompted an accelerated development effort described in Section 8.3, and has reignited calls for accelerated development of multivalent filovirus vaccines [4].

### Therapeutics and Monoclonal Antibodies

Alongside vaccination, the therapeutic management of Ebola virus disease has been transformed by the advent of monoclonal antibody (mAb) treatments. The landmark PALM (Pamoja Tulinde Maisha) randomised controlled trial, conducted during the 2018–2020 outbreak in eastern DRC, compared four investigational therapeutics and demonstrated a substantial survival benefit for two monoclonal antibody products [68]. On this basis, two mAb-based therapies subsequently received regulatory approval: Inmazeb (atoltivimab/maftivimab/odesivimab-ebgn, a three-antibody cocktail) and Ebanga (ansuvimab-zykl, a single monoclonal antibody) [68]. Among patients with low viral loads who received either agent, mortality fell markedly compared with the comparator arms, establishing early mAb administration as a new standard of care for Zaire ebolavirus infection [68]. These antibodies were raised against, and their clinical efficacy was demonstrated against, the Zaire ebolavirus glycoprotein; they are not licensed or clinically validated against Sudan virus or Bundibugyo virus, mirroring the species-specific licensing status of rVSV-ZEBOV, although in vitro and animal data on partial cross-reactivity for some candidates are being actively investigated (see Section 8.3). The absence of licensed species-specific therapeutics for the 2026 Bundibugyo outbreak at onset therefore compounded the vaccine gap described above, initially leaving responders reliant on supportive care and classic public health measures, before the launch of the PARTNERS platform trial described in Section 8.3.

## 7. Future Challenges and Global Preparedness

Future Ebola preparedness will depend largely on the ability of healthcare systems and international institutions to respond to increasingly interconnected global challenges. Climate change, ecological disruption, population growth, urbanization, forced displacement, and political instability are all expected to continue shaping the epidemiology of emerging infectious diseases over the coming decades [2,7]. The frequency and geographic range of filovirus outbreaks have shown an upward trend, and modelling of broader cross-species viral sharing suggests that climate-driven changes in bat distribution could alter contact patterns in new regions of sub-Saharan Africa by 2070 [44,45], though, as noted in Section 3, Ebola-specific projections of this kind remain hypothesis-generating rather than established.

Fragile healthcare systems remain particularly vulnerable to epidemic amplification. In many endemic regions, persistent limitations in laboratory capacity, surveillance infrastructure, infection prevention measures, and healthcare workforce availability continue to delay outbreak recognition and weaken response capacity [3,47,48], as introduced in Section 3 and Section 4. Humanitarian emergencies and armed conflicts may further compromise public health interventions and reduce trust in institutions; the 2018–2020 eastern DRC outbreak demonstrated that even with a highly effective vaccine, conflict dynamics can prolong an epidemic for nearly two years [23,30].

The increasing frequency of zoonotic threats has reinforced the importance of integrated preparedness frameworks combining epidemiological surveillance, genomic sequencing, ecological monitoring, mathematical modelling, and community-based response systems [20,48,69]. Early-warning systems and coordinated surveillance networks may become increasingly important for identifying outbreaks before large-scale amplification occurs; the Sentinel early-warning initiative and Africa CDC’s Integrated Disease Surveillance and Response (IDSR) framework represent steps in this direction, but gaps in real-time data sharing and interoperability remain [70,71].

Preparedness strategies must also address the long-term consequences of Ebola outbreaks. Survivor care, stigma reduction, mental health support, and monitoring for viral persistence (see Section 5) have become increasingly relevant components of post-outbreak management [24,34,48], necessitating long-term follow-up programmes for survivors [34,35].

### 7.1. The Role of Artificial Intelligence and Emerging Technologies

The technologies discussed in this subsection span a wide range of maturity, from retrospective computational studies to tools with documented, if still limited, field deployment. We have organised the discussion to keep this distinction explicit throughout, since capability demonstrated in a methodological paper is not equivalent to evidence of improved real-world outbreak outcomes.

#### 7.1.1. AI-Driven Predictive Modeling and Early Warning Systems

Artificial intelligence (AI) and machine learning (ML) approaches are being explored to improve real-time forecasting of EVD transmission. Unlike traditional compartmental models (e.g., Susceptible-Exposed-Infectious-Removed (SEIR)), which often assume static parameters, AI-driven frameworks can in principle integrate diverse data streams, including human mobility patterns, ecological niche variables, climate data, and real-time surveillance reports, to generate probabilistic risk maps and case forecasts. It is important to distinguish this predictive capability, demonstrated so far mainly in retrospective or simulated analyses, from evidence of improved real-world outbreak outcomes; prospective evidence that such models improve public health decision-making during an active filovirus outbreak is still lacking.

The DeepEVD framework, introduced by researchers in 2025, integrates human mobility data into a deep learning architecture based on spatio-temporal feature learning, and reported improved predictive performance over conventional models that neglect population movement in retrospective analyses [72]. Similarly, an artificial neural network (ANN)-based model has been developed to extend deterministic SEIR frameworks by incorporating a disability compartment [73], and Morlet wavelet neural networks (MWNN) optimized via hybrid genetic algorithm and adaptive simulated annealing have been applied to simulate EVD dynamics with non-linear incidence rates in modelling studies [74]. These are computational and methodological contributions; none has, to our knowledge, been prospectively validated as an operational forecasting tool during a live filovirus outbreak.

Beyond transmission modeling, AI is increasingly used for epidemic intelligence and early warning for infectious diseases in general. A 2026 review in Archives of Computational Methods in Engineering discusses the growing role of AI in epidemic modeling and pandemic preparedness, noting that event-based surveillance systems such as HealthMap, BlueDot, and EPIWATCH have detected unusual health events from online sources ahead of official alerts for other pathogens [75], most notably BlueDot’s early flagging of atypical pneumonia in Wuhan in December 2019 [76]. This is evidence of general epidemic-intelligence capability rather than of a validated Ebola-specific or Bundibugyo-specific forecasting tool; we did not identify a peer-reviewed evaluation of these platforms’ performance during a filovirus outbreak specifically. The Coalition for Epidemic Preparedness Innovations (CEPI) has described a “Pandemic Preparedness Engine”, a generative AI platform intended to scan global data streams, identify emerging pathogens with pandemic potential, and propose antigen designs for rapid vaccine development [77]; as of this writing it remains under development, and its operational performance has not yet been independently evaluated.

#### 7.1.2. Field-Deployable Genomic Surveillance and Digital Technologies

Portable sequencing technologies have improved real-time pathogen surveillance with a stronger evidence base than the predictive-modelling tools above. Oxford Nanopore Technologies’ MinION device, a long-read sequencer the size of a USB memory stick, was first deployed during the 2014–2016 West African Ebola epidemic, enabling researchers to generate viral genome sequences within 24 h of sample collection in a mobile laboratory in Guinea [16,51], allowing real-time reconstruction of transmission chains. A 2025 review in Current Clinical Microbiology Reports notes that long-read sequencing has since been used for rapid pathogen identification in field settings during both Ebola and COVID-19 outbreaks [16]. Despite these advances, major disparities persist: during the 2018–2020 eastern DRC outbreak, genomic sequencing capacity was supported almost entirely by external actors [3], and over 50% of sub-Saharan African countries lack adequate laboratory capacity for routine genomic surveillance, with inequitable coverage disproportionately affecting rural, displaced, and conflict-affected populations [78].

Unmanned aerial vehicles (UAVs) have documented operational use for sample transport and vaccine delivery. In the DRC, drones capable of traveling up to 130 km carrying 3 kg of cargo have been used to transport Ebola test samples from hard-to-reach health zones to reference laboratories, reducing turnaround time from days to hours [79]. VillageReach, in collaboration with provincial health authorities, has established drone networks covering over 37,000 km^2^ in Équateur Province [79], and similar systems are operational in Rwanda, where drones serve approximately 450 health facilities and a population of over 11 million people [80,81].

Blockchain technology has been proposed to enhance supply chain transparency and vaccine traceability. The SecureVAX system, a blockchain-enabled vaccine passport platform, and a separate blockchain-based immunization record-keeping technique have been described in the literature [82,83]; at present these are conceptual or small-scale prototype systems rather than deployed outbreak infrastructure, and we are not aware of a blockchain platform that has been operationally used during an Ebola or Bundibugyo virus response. Lightweight permissioned implementations for emergency-response logistics remain at an early piloting stage.

Mobile health (mHealth) applications, particularly chatbots, have documented operational use for risk communication and community engagement [84]. In June 2026, Kenya’s Ministry of Health launched “JALI”, a WhatsApp-based chatbot providing verified Ebola information and continuous engagement to counter misinformation [85]. During the West African Ebola epidemic, voice-based, multilingual mobile applications were deployed to spread reliable public health information via peer-to-peer sharing, reaching populations with low literacy [86], and BBC Media Action’s Welbodi Tok platform, originally developed during that outbreak, has since been adapted for malaria and other health threats [87].

#### 7.1.3. Integrated Preparedness Frameworks

The technologies discussed above range considerably in evidentiary maturity, from proposed or prototype systems (blockchain traceability, several AI forecasting models) to tools with genuine operational track records (portable genomic sequencers, drone logistics networks, mHealth chatbots). None alone provides a comprehensive solution: a predictive AI model is of little value if it cannot be integrated with real-time surveillance data; a drone delivery network is ineffective without reliable cold chain infrastructure at the receiving end; a blockchain vaccine tracker cannot prevent diversion if local health facilities lack trained personnel to verify doses; and a mobile health chatbot cannot counter misinformation if community trust in health authorities is already eroded. These technologies function, at best, as force multipliers that amplify the effectiveness of core public health interventions when embedded within a coherent, systems-level preparedness framework, rather than as standalone fixes.

This understanding has led to the development of integrated preparedness frameworks that position digital technologies and AI as cross-cutting enablers rather than isolated innovations, recognising that outbreaks emerge from the intersection of ecological, social, and healthcare-system factors and that responses must address all three domains simultaneously. The recognition of filoviruses as prototype pathogens for future epidemic threats, as articulated by the WHO R&D Blueprint and CEPI, further emphasises the need for strategies that combine surveillance systems, rapid diagnostics, vaccine platforms, resilient healthcare infrastructures, and coordinated international response mechanisms [36,47,69].

For these reasons, the concept of One Health has become increasingly central to future filovirus preparedness. Ebola virus disease should no longer be viewed solely as a viral hemorrhagic fever, but also as an indicator of the interaction between environmental change, societal vulnerability, ecological disruption, and global health security. The One Health approach, integrating human, animal, and environmental health surveillance, is increasingly regarded as a strategic necessity, given the repeated association between land-use change and Ebola spillover events [8,11].

**Figure 3:**
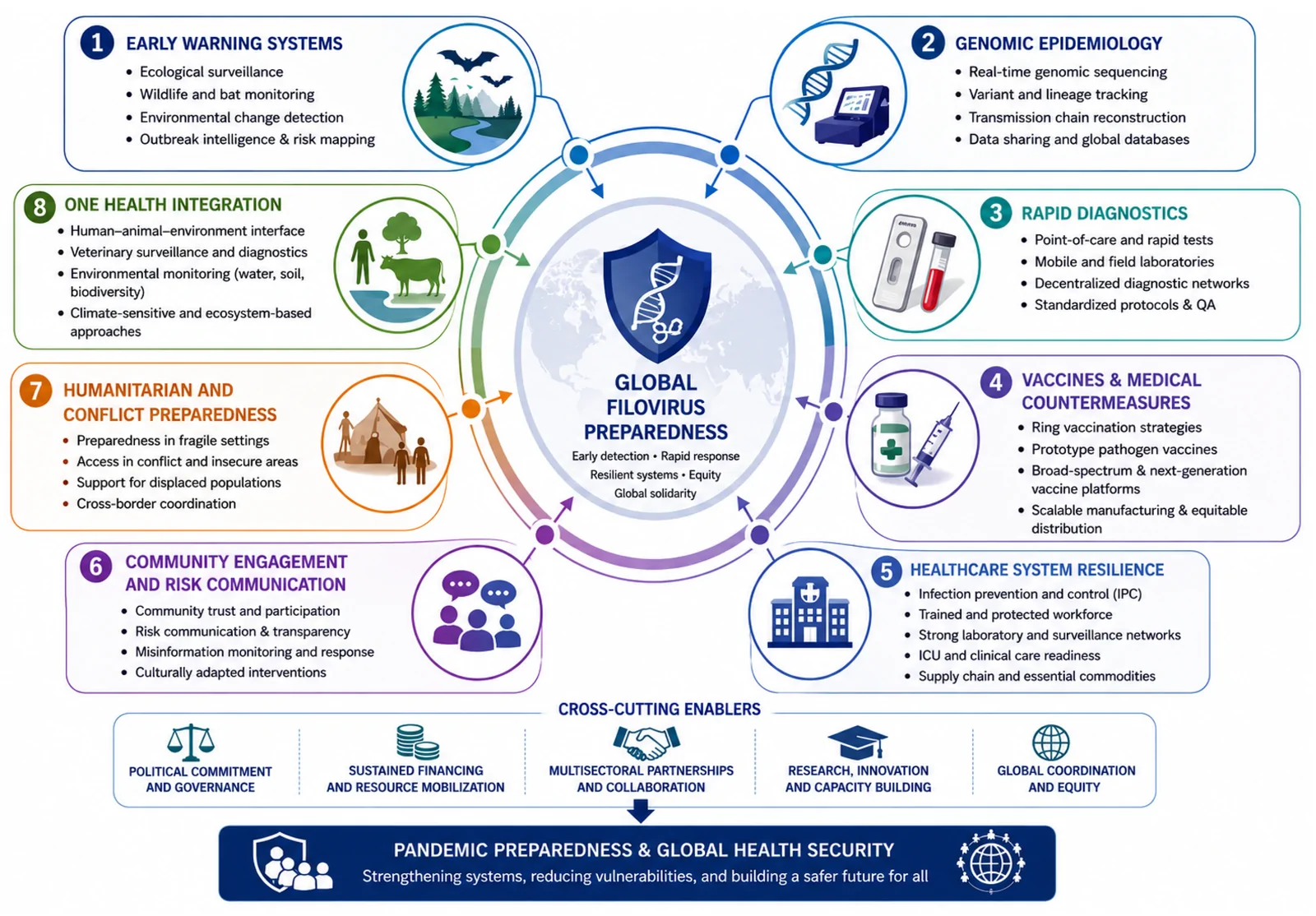
Integrated preparedness framework for future filovirus threats within a One Health and global health security perspective, showing eight radial preparedness domains and cross-cutting enablers linked through bidirectional feedback relationships.

Figure 3 synthesises these components into a single conceptual framework for future filovirus threats, viewed through a modern One Health and global health security lens. The figure is organised around a central hub (“Global Filovirus Preparedness”) surrounded by eight numbered radial domains: (1) early warning systems (ecological surveillance, wildlife and bat monitoring, environmental change detection, outbreak intelligence); (2) genomic epidemiology (real-time sequencing, variant and lineage tracking, transmission chain reconstruction, data sharing); (3) rapid diagnostics (point-of-care and rapid tests, mobile and field laboratories, decentralised diagnostic networks, standardized protocols); (4) vaccines and medical countermeasures (ring vaccination strategies, prototype pathogen vaccines, broad-spectrum and next-generation platforms, scalable manufacturing and equitable distribution); (5) healthcare system resilience (IPC, trained and protected workforce, laboratory and surveillance networks, ICU and clinical care readiness, supply chain and essential commodities); (6) community engagement and risk communication (trust and participation, transparency, misinformation monitoring, culturally adapted interventions); (7) humanitarian and conflict preparedness (preparedness in fragile settings, access in conflict and insecure areas, support for displaced populations, cross-border coordination); and (8) One Health integration (human–animal–environment interface, veterinary surveillance and diagnostics, environmental monitoring, climate-sensitive and ecosystem-based approaches). This circle of eight domains is underpinned by a horizontal strip of cross-cutting enablers: political commitment and governance; sustained financing and resource mobilization; multisectoral partnerships and collaboration; research, innovation, and capacity building; and global coordination and equity, feeding into the overarching goal of pandemic preparedness and global health security.

The bidirectional arrows shown in the figure connect the eight radial domains to the central hub and to one another, illustrating the feedback loops described in the figure caption: for example, weak healthcare system resilience (domain 5) exacerbates healthcare-associated transmission, which in turn increases the burden on already fragile facilities; community mistrust (domain 6) reduces acceptance of public health measures, amplifying transmission and delaying case detection; and conversely, strong community engagement accelerates vaccine acceptance (domains 6 → 4), robust genomic surveillance informs R&D priorities (domains 2 → 4), effective international coordination enables equitable vaccine distribution (cross-cutting enablers → 4), and digital technologies enhance early warning and logistics (domains 1, 2 → 7).

Together, these interconnected components illustrate that no single intervention can ensure preparedness. Rather, a systems approach that adapts to local context, learns from each outbreak, and continuously invests in both technical and social infrastructure is required. The framework is not static, evolving with emerging threats, new technologies, and shifting geopolitical landscapes.

## 8. Discussion

This narrative review has traced the evolving epidemiology of Ebola virus disease through the lens of One Health and global preparedness, highlighting persistent gaps and emerging opportunities. Several key findings emerge from our synthesis. First, the recognized range of Ebola outbreak contexts has broadened over time, from predominantly rural spillovers to episodes additionally shaped by ecological disruption, population mobility, conflict, and healthcare fragility, without rural zoonotic spillover ceasing to be the predominant mode of emergence. Second, genomic surveillance and real-time sequencing have improved outbreak investigation, yet their benefits remain unequally distributed, with many endemic regions lacking sustainable infrastructure. Third, the absence of licensed vaccines and therapeutics for Sudan and Bundibugyo virus disease remains a major vulnerability; a first-ever clinical efficacy trial of a Sudan virus vaccine candidate began in Uganda in February 2025, and Bundibugyo-specific candidates only entered clinical evaluation during the 2026 outbreak. Fourth, artificial intelligence and digital technologies offer promising tools for early warning, logistics, and community engagement, but the evidence base for their real-world operational impact during filovirus outbreaks specifically remains limited, and deployment in low-resource settings faces substantial barriers. Fifth, the 2026 Bundibugyo outbreak has again illustrated that structural weaknesses in surveillance funding, response timeliness, and community trust continue to recur.

### 8.1. Lessons from COVID-19 for Ebola Preparedness

The COVID-19 pandemic reshaped global preparedness frameworks for emerging infectious diseases and renewed attention to surveillance systems, rapid diagnostics, resilient healthcare infrastructures, and coordinated international responses. Several strategies that later became central during the COVID-19 response had already been developed, tested, or refined during previous Ebola outbreaks, particularly in West and Central Africa [88,89]. Experiences gained during Ebola epidemics highlighted the importance of rapid case identification, contact tracing, isolation procedures, community engagement, and risk communication, which subsequently became core components of COVID-19 containment strategies worldwide; Ebola outbreaks also demonstrated how misinformation, stigma, and mistrust toward institutions can undermine public health interventions, reinforcing the importance of transparent, culturally adapted engagement [24,25].

Another major lesson concerns healthcare system resilience. Both Ebola and COVID-19 exposed the vulnerability of fragile healthcare infrastructures and showed how healthcare-associated transmission can rapidly amplify epidemics when infection prevention measures are insufficient [19,48]. The COVID-19 era also accelerated the implementation of digital technologies and integrated surveillance systems; digital contact-tracing platforms, real-time epidemiological dashboards, mobile health applications, genomic surveillance infrastructures, and modelling tools became increasingly relevant during responses to both Ebola and COVID-19 [18,20,71], although important disparities in infrastructure, connectivity, and technological access remain.

Perhaps the most important lesson emerging from both Ebola and COVID-19 is that preparedness cannot rely exclusively on reactive outbreak control. Sustainable preparedness requires continuous investment in surveillance systems, laboratory networks, genomic sequencing capacity, healthcare workforce training, vaccine infrastructure, and international collaboration, particularly in regions characterized by recurrent epidemic threats and fragile healthcare systems [3,33,47,70]. The growing convergence between Ebola preparedness and broader pandemic preparedness frameworks reflects a changing global health perspective in which emerging infectious diseases are increasingly viewed as interconnected threats shaped by ecological disruption, globalization, climate-sensitive environmental changes, and healthcare system resilience.

### 8.2. Barriers to Implementing AI and Digital Technologies in Low-Resource Settings

Despite the promise of AI, portable sequencing, drones, blockchain, and mobile health tools, their deployment in low-resource settings faces substantial and often interconnected barriers. A 2026 analysis of epidemiological surveillance in sub-Saharan Africa identifies persistent limitations: lack of reliable internet connectivity in rural areas, limited digital literacy among both health workers and communities, high upfront and maintenance costs, fragmented and siloed surveillance systems, and weak specimen referral networks [78]. These gaps are compounded by unreliable electricity supply, which can render cold chain monitoring and sequencing equipment inoperable without solar or battery backups that are often unavailable or too expensive [90].

Digital divides are not only technical but also social. Low digital literacy means that even when smartphones are available, community health workers may struggle to use complex applications, and communities may not trust or understand chatbot-based risk communication. Language barriers further limit the reach of English-centric platforms, since most digital health tools rely on dominant languages, excluding millions who speak low-resource African languages [91]. Affordability remains a critical barrier to mobile internet adoption in Sub-Saharan Africa. According to “The Mobile Economy Africa 2026” report [92], the cost of an entry-level internet-enabled handset in the region represents on average 24% of monthly income in low- and middle-income countries, and can rise to as much as 80% for the poorest population segments. This far exceeds the affordability threshold recommended by the UN Broadband Commission for entry-level broadband services (2% of monthly income), highlighting the structural economic constraints that limit digital participation. Furthermore, the report identifies the usage gap, the proportion of the population covered by mobile broadband but not yet online, as Africa’s defining digital challenge, with 63% of the population remaining unconnected despite network availability.

Concerns about data privacy, algorithmic bias, and informed consent must be addressed through co-design with local communities, investment in digital infrastructure, and the development of open-source, offline-capable solutions. Algorithmic bias is a particular risk: if training data over-represent urban, wealthier, or better-connected populations, predictive models may systematically under-detect outbreaks in rural, displaced, or marginalised groups [93]. During the 2018–2020 DRC Ebola outbreak, rumours that digital contact tracing was being used for political surveillance fuelled community mistrust, undermining uptake of mobile health tools [61].

One Health platforms that integrate human, animal, and environmental surveillance, such as the MOBILISE mobile laboratory system and proposals for AI-assisted emergency operations centre software, have been developed and piloted in other contexts to help predict outbreak hotspots and allocate resources proactively [91,92]; we did not identify documentation of their deployment specifically within the current Bundibugyo response (see Section 8.3). More broadly, such pilots often struggle to scale due to fragmented ownership across ministries, lack of interoperability standards, and short-term donor funding cycles; sustainability requires embedding digital tools within national health information systems (e.g., DHIS2) and securing multi-year government and development bank commitments. When properly implemented with community engagement and robust governance, AI and emerging technologies can serve as force multipliers that enhance early warning, accelerate logistics, support behaviour change, and reduce the burden on frontline responders, rather than as substitutes for strong health systems. More broadly, persistent underinvestment in basic public health infrastructure, i.e., laboratory networks, cold chains, trained personnel, and community trust, remains the single greatest obstacle to effective filovirus preparedness; without addressing these foundational gaps, even the most sophisticated technologies will remain pilots or donor-dependent demonstrations rather than standard components of outbreak response.

### 8.3. The 2026 Bundibugyo Outbreak as a Contemporary Preparedness Challenge

The ongoing 2026 Bundibugyo Ebola outbreak in the DRC further illustrates how Ebola virus disease continues to challenge global preparedness frameworks despite the progress achieved after the West African epidemic. First confirmed in mid-May 2026, the outbreak has since spread across Ituri, North Kivu and South Kivu provinces in the DRC, affecting remote, forested areas with limited road access and intermittent security; cases were also confirmed in Uganda, where the outbreak was declared over on 28 July 2026. Because the outbreak in the DRC is evolving rapidly, we report the most recent figures available at the time of this revision rather than a single fixed snapshot, and we note that these figures are expected to change further before publication: WHO and Africa CDC situation reports through early June 2026 documented several hundred confirmed cases, rising through late June to over 1150 confirmed cases and around 300 deaths (CFR approximately 26%) across the DRC, Uganda, and an imported case in France, and by early July to roughly 1480 confirmed cases and over 450 deaths [38,94,95]. As of 9 August 2026, the DRC had reported 4381 confirmed cases and 2011 confirmed deaths [96], with further imported cases identified among healthcare workers evacuated to Germany and among travellers to France [97]. Because this outbreak is moving rapidly (ECDC reported 4665 confirmed cases and 2184 deaths through 12 August 2026 [38]), these figures should be updated one final time immediately prior to publication. As in the introduction to this review, we emphasise that these figures are almost certainly underestimates of true transmission, given under-reporting, insecurity, and population displacement in the affected provinces.

Recent events have again highlighted the vulnerability of regions affected by armed conflict, population displacement, fragile healthcare systems, and limited surveillance infrastructure, all of which can contribute to delayed outbreak detection and sustained transmission [3,4,5,6]. The epicentre in the DRC lies in a similarly complex environment to that which hosted the 2018–2020 eastern DRC outbreak: numerous armed groups operate in the area, frequent population movements cross porous borders, and community distrust of authorities remains high, conditions that have been reported to delay contact tracing and safe burial activities and allow secondary transmission chains to establish [3,6]. Reports have also described attacks on and displacement of healthcare workers and facilities during the response, though we have not identified a single authoritative, continuously updated count of healthcare-worker deaths or facility closures specific to this outbreak, and we therefore describe this qualitatively rather than with a specific figure.

The outbreak remains active in the DRC, with linked cases previously reported in Uganda; Uganda’s Ministry of Health declared its national outbreak over on 28 July 2026 [98], following 20 confirmed cases and two deaths and a 42-day period without a new locally transmitted case, while cross-border surveillance and a continued DRC–Uganda border closure remain in place.

The current outbreak has also exposed persistent gaps in filovirus preparedness. At outbreak onset, no licensed vaccine or therapeutic existed for Bundibugyo virus disease, and diagnostic testing initially relied heavily on sample transport to centralized laboratories. This gap has since narrowed: WHO added the first molecular diagnostic for Bundibugyo virus to its Emergency Use Listing on 2 July 2026, and testing capacity across DRC/Uganda laboratories subsequently expanded substantially. On the vaccine side, the University of Oxford, in partnership with the Serum Institute of India (SII) and with funding from CEPI, launched the first-in-human trial of a ChAdOx1-vectored Bundibugyo vaccine (ChAdOx1 BDBV) in July 2026, evaluating safety and immunogenicity in 50 healthy adults aged 18–55; this vaccine uses the same viral-vector platform as the Oxford/AstraZeneca COVID-19 vaccine, and SII has manufactured and stockpiled several hundred thousand investigational doses in anticipation of future need [38]. On the therapeutics side, the WHO-sponsored PARTNERS (Platform Adaptive Randomised Trial for New and Repurposed Filovirus TreatmentS) trial began enrolling patients in the DRC in July 2026, coordinated by the Institut National de Recherche Biomédicale (INRB), the Institute of Tropical Medicine (Belgium), and the University of Oxford, in collaboration with Africa CDC; it is evaluating the monoclonal antibody MBP134 and the antiviral remdesivir, alone and in combination, as the first randomized evaluation of treatments specifically for Bundibugyo virus disease [95]. An independent WHO expert panel convened in late May 2026 additionally prioritized the monoclonal antibody maftivimab, given its existing field availability in the DRC, and identified the oral antiviral obeldesivir as a priority candidate for post-exposure prophylaxis, on the basis of animal studies in which once-daily obeldesivir given for 10 days provided up to 100% protection in monkeys against Zaire and Sudan virus challenge when treatment began 24 h after exposure; WHO noted that its effectiveness specifically against Bundibugyo virus in humans, and the operational feasibility of post-exposure prophylaxis given ongoing contact-tracing challenges, remain to be established [37].

Consequently, response teams have continued to rely heavily on classic public health measures, i.e., case finding, isolation, contact tracing, safe burials, and community engagement, which, while effective when rigorously implemented, are far more difficult to sustain in conflict-affected zones, alongside the newly initiated vaccine and therapeutic trials described above. Tools such as mobile field-laboratory platforms (e.g., the MOBILISE system) and AI-assisted emergency operations centre software have been developed and piloted elsewhere and represent plausible components of a future response; we did not identify documentation of their deployment in the current Bundibugyo outbreak specifically, and reference to their use in this outbreak should therefore be treated as aspirational rather than established [99,100].

More broadly, the 2026 outbreak illustrates how the epidemiology of filovirus diseases can become intertwined with geopolitical instability, ecological disruption, healthcare fragility, and regional mobility patterns when these factors coincide. In this context, preparedness is best viewed as a continuous process rather than a response activated only during emergencies; sustained investment in surveillance capacity, laboratory infrastructure, regional coordination, and public-health resilience will likely remain essential to limit the impact of future outbreaks. Box 1 synthesises the key lessons from past and present Ebola outbreaks to inform future pandemic preparedness.

Box 1 **Key Lessons from Ebola for Future Pandemic Preparedness.**

- Importance of rapid outbreak detection and integrated surveillance systems
- Central role of community trust and risk communication
- Need for resilient healthcare systems and IPC capacity
- Value of genomic epidemiology and real-time sequencing
- Importance of ecological surveillance within a One Health framework
- Persistent challenges related to vaccine equity and global access
- Role of epidemiological modelling and early-warning systems
- Need for preparedness strategies adapted to humanitarian and conflict settings
- Recognition of viral persistence and survivor follow-up as emerging preparedness priorities
- Increasing relevance of filoviruses as prototype pathogens for future epidemic threats
- Integration of artificial intelligence and digital technologies for early warning, logistics, and community engagement

Despite these advances, several knowledge gaps continue to constrain filovirus preparedness and should be prioritised in future research. First, the natural reservoir of ebolaviruses remains formally unconfirmed, and the precise ecological triggers of spillover are only partially understood, limiting the predictive value of ecological surveillance. Second, the durability of vaccine-induced immunity, the potential for cross-protection across ebolavirus species, and optimal deployment strategies in conflict-affected settings remain incompletely defined. Third, until mid-2026, licensed vaccines and species-specific therapeutics were entirely lacking for Sudan and Bundibugyo viruses; even with the trials described above now underway, the clinical determinants of viral persistence and late recrudescence in survivors, and the resulting transmission risk, require longitudinal study. Fourth, the real-world effectiveness, equity, and scalability of AI-driven early-warning systems and digital tools in low-resource settings have yet to be rigorously evaluated. Addressing these gaps will require sustained investment in basic ecological and virological research, multi-country survivor cohorts, pan-filovirus countermeasure development, and pragmatic implementation studies conducted where outbreaks actually occur.

## 9. Conclusions

The epidemiology of Ebola virus disease has evolved from a picture dominated by isolated rural outbreaks to one encompassing a broader, more complex range of contexts and amplification mechanisms, challenging traditional preparedness paradigms. This review has traced that evolution through successive epidemics, from 1976 to the ongoing 2026 Bundibugyo crisis, and identified persistent structural weaknesses that continue to affect response efforts despite decades of lessons. Three overarching conclusions emerge.

First, no single intervention, whether a vaccine, a diagnostic test, or a digital tool, can ensure outbreak containment. Effective preparedness requires systems thinking: integrating ecological surveillance, genomic epidemiology, resilient healthcare infrastructure, community engagement, and international coordination under a One Health framework. The 2026 outbreak has reaffirmed that where these components are weak, even advanced biomedical tools cannot compensate.

Second, the digital transformation of outbreak response offers real, if unevenly demonstrated, opportunities for early warning, logistics, and risk communication. Portable sequencing, drone logistics, and mHealth platforms have documented operational track records; several AI forecasting and blockchain applications remain earlier-stage or largely conceptual, and their potential contribution should be assessed against this evidence, not assumed. Realising this potential, where it exists, will require addressing persistent barriers, e.g., unreliable electricity, high data costs, digital illiteracy, fragmented ownership, and short-term funding cycles, through sustained investment, local co-design, and integration into national health information systems.

Third, the global community must move from episodic, reactive emergency funding to predictable, multi-year financing for core capacities: laboratory networks, cold chains, trained healthcare workers, survivor follow-up programmes, and community trust-building. The inequities that have historically left Sudan and Bundibugyo viruses without licensed vaccines or therapeutics, while Zaire ebolavirus has multiple licensed countermeasures, reflect a moral and strategic gap that the February 2025 Sudan virus vaccine efficacy trial in Uganda and the newly launched ChAdOx1 BDBV vaccine trial and PARTNERS therapeutics trial for Bundibugyo virus begin to address, but which will require diversified vaccine platforms and regional manufacturing to close durably.

The 2026 Bundibugyo outbreak is not an exception but a plausible harbinger of future filovirus threats in an era of ecological disruption, climate change, and geopolitical instability. Whether its lessons translate into lasting structural change depends on the collective will of national governments, multilateral agencies, donors, and researchers. Preparedness is not a state to be achieved but a continuous process of learning, adapting, and investing before the next alarm sounds.

## Figures and Tables

**Table 1:** Major Ebola outbreaks and key epidemiological characteristics. Overview of major Ebola virus disease outbreaks and their evolving epidemiological features. The table summarizes the principal outbreak settings, dominant transmission characteristics, and key preparedness lessons that contributed to the modern understanding of filovirus epidemiology and outbreak response.

Year	Country/Region	Ebola Species	Estimated Cases/Deaths	Major Epidemiological Characteristics	Key Preparedness Lessons
1976	Sudan and Zaire (now DRC)	Sudan virus/Zaire ebolavirus	First recognized outbreaks; high CFR	Rural transmission, limited healthcare infrastructure, nosocomial amplification	Recognition of EVD as major emerging pathogen; importance of IPC [1]
1995	Kikwit, DRC	Zaire ebolavirus	Major urban-associated outbreak with high mortality	Healthcare-associated transmission, delayed outbreak recognition, inadequate IPC	Need for rapid isolation, laboratory capacity, healthcare worker protection [19,28]
2000–2001	Uganda	Sudan virus	Large outbreak with improved international response	Enhanced surveillance, contact tracing, coordinated response	Importance of international collaboration and outbreak coordination [32]
2013–2016	Guinea, Sierra Leone, Liberia	Zaire ebolavirus	Largest Ebola outbreak (>28,000 cases)	Urban transmission, cross-border spread, fragile health systems, misinformation	Strengthen surveillance, rapid diagnostics, community engagement, global preparedness [1,30,31]
2018–2020	Eastern DRC	Zaire ebolavirus	Prolonged outbreak in conflict-affected settings	Armed conflict, population displacement, vaccination deployment, genomic surveillance	Vaccination strategies, genomic epidemiology, preparedness in humanitarian settings [14,20,23]
2022	Uganda	Sudan virus	Re-emergence in resource-limited settings	No licensed Sudan vaccine, rapid outbreak spread, healthcare fragility	Need for broader filovirus vaccine platforms and strengthened preparedness [32,33]
2026	DRC and Uganda	Bundibugyo virus	Ongoing cross-border outbreak, public health emergency	Cross-border transmission, fragile surveillance, humanitarian complexity, no Bundibugyo-specific vaccines	One Health preparedness, rapid diagnostics, regional coordination, diversified vaccine development [3,4,5,6,36,37,38]

**Table 2:** Ecological, societal, and healthcare-related drivers contributing to Ebola emergence and outbreak amplification.

Category	Driver	Association with Outbreaks	Consequence	Preparedness Implication
Ecological	Deforestation/ ecosystem fragmentation	Associated with increased human–wildlife interaction	Higher zoonotic spillover risk	Ecological surveillance and One Health integration [2,7,8,10]
Ecological	Mining and environmental disruption	Associated with habitat alteration and displacement	Changing spillover dynamics	Environmental monitoring, occupational preparedness [11]
Climate-related	Climate-sensitive environmental changes	Associated with altered wildlife migration and food availability	Possible modification of emergence/transmission patterns	Climate-informed surveillance and ecosystem monitoring [2]
Biological	Viral persistence in survivors	Documented in immune-privileged sites	Recrudescence-associated outbreaks	Long-term survivor monitoring and genomic surveillance [15,34,35]
Social	Population mobility/cross-border movement	Associated with rapid dissemination	Cross-border transmission, delayed containment	Regional coordination, border preparedness [1,20]
Social	Misinformation, stigma, mistrust	Associated with reduced adherence to interventions	Delayed detection, outbreak amplification	Risk communication, community engagement [24,25]
Humanitarian	Armed conflict and displacement	Disrupts healthcare delivery and surveillance	Persistent transmission chains	Preparedness for conflict-affected settings [11,23,30]
Healthcare-related	Weak IPC infrastructure	Associated with nosocomial amplification	Increased transmission among patients/staff	Strengthened IPC and worker protection [19,28]
Healthcare-related	Limited laboratory/surveillance capacity	Associated with delayed diagnosis	Prolonged undetected transmission	Investment in diagnostic and surveillance infrastructure [3,47]
System-level	Fragile healthcare systems	Associated with reduced outbreak response capacity	Epidemic amplification, increased mortality	Strengthening resilient healthcare systems [47,48]

## Data Availability

No new data were created or analyzed in this study. Data sharing is not applicable to this article.
